# Women’s autonomy and utilization of maternal healthcare in India: Evidence from a recent national survey

**DOI:** 10.1371/journal.pone.0243553

**Published:** 2020-12-09

**Authors:** Dinabandhu Mondal, Suranjana Karmakar, Anuradha Banerjee

**Affiliations:** 1 Centre for the Study of Regional Development, School of Social Sciences, Jawaharlal Nehru University, New Delhi, India; 2 Department of Women’s Studies, The University of Burdwan, Bardhaman, West Bengal, India; ESIC Medical College & PGIMSR, INDIA

## Abstract

**Objective:**

The present study aims to examine the association between women’s decision-making autonomy and utilization of maternal healthcare services among the currently married women in India.

**Methods:**

A total of 32,698 currently married women aged 15–49 years who had at least one live birth in the past five years preceding the survey and had information regarding autonomy collected by the National Family Health Survey 2015–16 were used for analysis. Bivariate and multivariate logistic regression models were employed for the analyses of this study.

**Results:**

Utilization of maternal healthcare services was higher among the women having a high level of decision-making autonomy compared to those who had a low autonomy in the household. The regression results indicate that women’s autonomy was significantly associated with increased odds of maternal healthcare services in India. Women with high autonomy had 37% and 33% greater likelihood of receiving ANC (AOR: 1.37, 95% CI: 1.25–1.50) and PNC care (AOR: 1.33, 95% CI: 1.24–1.42) respectively compared to women having low autonomy. However, no significant association was observed between women’s autonomy and institutional delivery in the adjusted analysis.

**Conclusion:**

This study recommends the need for comprehensive strategies involving improvement of women’s autonomy along with expansion of education, awareness generation regarding the importance of maternity care, and enhancing public health infrastructure to ensure higher utilization of maternal healthcare services that would eventually reduce maternal mortality.

## Introduction

Maternal mortality remains a major public health concern in the world, especially in low and lower-middle-income countries. As per the recent World Health Organization estimates, around 229 thousand women died in 2017 due to pregnancy-related complications and childbirth. Approximately 66% and 20% of global maternal deaths occurred in Sub-Saharan Africa and South Asia, respectively [1]. In 2015, United Nations General Assembly set a target under Sustainable Development Goals (SDG-3.1) to reduce global maternal mortality to less than 70 per 100,000 live births by 2030. In India, although there has been a significant improvement in bringing down the maternal mortality rate (MMR) from 370 in 2000 to 145 per 100,000 live births in 2017, the country reports one of the highest numbers of maternal mortality (35,000) worldwide. To achieve the SDG goal in India, substantial progress is still required to address the obstetric causes of maternal mortality, as studies indicate that almost 73% of all maternal deaths between 2003 and 2009 were due to direct obstetric causes [2]. Inadequate utilization of maternal healthcare services is a significant contributor to high maternal morbidity and mortality [3, 4]. Proper access to maternal healthcare—antenatal care (ANC), delivery care, and postnatal care (PNC) is important for the health of mothers as well as their offsprings. It is reported that maternal care utilization among Indian women is not adequate. Only 51% women received four or more ANC visits, 21% had full ANC care, 79% had delivery in a health facility, and 65% had PNC check-ups within two days of delivery [5].

Women’s autonomy is a multidimensional concept indicating control over the resources and ideologies; it implies self-confidence and inner transformation of one’s consciousness to overcome external barriers or traditional ideologies [6]. Autonomy is usually measured by three dimensions: access to and control over resources, participation in decision-making, and freedom of movement [7, 8]. Several studies conducted in Bangladesh [9, 10], Nepal [11], Ethiopia [12], Nigeria [13], and Ghana [14] documented that women’s autonomy has a positive impact on maternal healthcare utilization. These studies indicate that when women’s autonomy is restricted by limiting their movement, involvement in decision-making and financial independence, the consequences are relatively lower utilization of reproductive care services among women.

Although a number of previous studies have examined socio-economic, demographic, and accessibility-related factors for utilization of maternity care in India [15–17], the influence of women’s autonomy on access to reproductive health services has gained poor attention in this country. In the backdrop of a higher incidence of maternal mortality in India, it is of paramount importance to explore the link between women’s autonomy and utilization of maternity care services using a nationally representative dataset. It would enhance one’s understanding of the pathways to reproductive health and provide a new perspective for policy interventions to address maternal healthcare needs in the country. The present study attempts to examine the association between women’s autonomy and utilization of maternal healthcare services among the currently married women in India using the most recent data of the National Family Health Survey, 2015–16. The salient hypothesis of the study entails that women’s autonomy increases the likelihood of receiving maternal healthcare services.

## Methods

### Data source

The study is based on the latest Demographic and Health Survey in India, i.e. National Family Health Survey (NFHS-4) dataset conducted in 2015–16. The NFHS-4 is a nationally representative large-scale sample survey comprising 601,509 households, 699,686 women aged 15–49 years with a response rate of 97%, and 112,122 men aged 15–54 years with a response rate of 92%. The survey provides information on household characteristics, reproductive and child healthcare utilization, fertility, mortality, family planning methods, attitude, and behavior regarding HIV/AIDS, women empowerment, and health outcomes. The survey was carried out across all the states and union territories (UTs) in India and provided information up to the district level. The two-stage stratified cluster sampling technique was used to select the samples. Based on the 2011 Indian Census enumeration, 28,586 clusters (i.e., 8,397 in urban, 20,059 in rural areas, and 130 in slums) were selected in the first stage using probability proportional to size (PPS) method. The slums were selected from the list provided by Municipal Corporation Offices (MCOs). In the second stage, a complete household mapping and listing were done in the selected cluster and 22 households were selected systematically in each cluster from the household listing. A detailed description of the sampling design and survey procedure is stated in the national report of NFHS-4 [5].

### Study design and sample size

A cross-sectional study design was adopted in this study using the most recent data of the National Family Health Survey-4. We used the individual level data from the women file of NHFS-4. It provides the updated information about the women with regards to the demographic characteristics, utilization of maternal care services, women’s status and empowerment, nutritional status and anaemia, and domestic violence. The NFHS-4 interviewed 699,686 ever-married women aged 15–49 years. Among them, 190,898 ever-married women had at least one live birth in the past five years preceding the survey. However, information regarding women’s autonomy was available for 32,698 currently married women. Therefore, our analysis focused on the 32,698 currently married women aged 15–49 years who had at least one live birth in the past five years before the survey.

### Outcome variables

The dependent variables of this study are three indicators related to utilization of maternal healthcare services—antenatal care (ANC), delivery care, and postnatal care (PNC). ANC conceived as the primary care during pregnancy is generally estimated by the number of ANC visits and specific care provided during the visits [18]. Full antenatal care, defined as at least four antenatal visits, at least one tetanus toxoid (TT) injection and iron folic acid tablets or syrup taken for 100 or more days, was considered an indicator of ANC [5]. For delivery care, institutional delivery which is considered as ‘safe delivery’ was taken in this study. PNC check-ups of mothers by skilled personnel within 48 hours of delivery were considered as recommended by the Ministry of Health and Family Welfare (MoHFW), Government of India [5]. For empirical analysis, all these three variables—full ANC, institutional delivery, and PNC within 48 hours were dichotomized into binary variables (‘0’ indicating no and ‘1’ indicating yes).

### Predictor variables

Women’s decision-making autonomy is the key predictor in this study. Autonomy was defined as the decision-making authority in the household related to four components: own healthcare, household purchases, spending of money, and freedom of movement [17]. This information was collected by four questions asked to the women during the survey of NFHS-4: *i) person who usually decides on respondent’s healthcare*, *ii) person who usually decides on large household purchases*, *iii) person who usually decides what to do with money husband earns*, *and iv) person who usually decides on visits to family or relatives*. For each of the questions, the responses were captured by five options, such as respondent alone, respondent and partner together, partner alone, someone else, and others. These variables were assigned as ‘1’ in the first two responses where women’s involvement in decision-making was reported and the rest as ‘0’ indicating no role of women. Adding these four variables, a final score, ranging from 0 to 4, was generated and categorized into three groups of decision-making autonomy. Women who had scores of four were considered as having high overall decision-making autonomy, women with scores of 1 to 3 as medium autonomy, and women with score 0 as low autonomy.

### Confounding variables

In addition to women’s autonomy, several other socio-demographic variables were also considered as confounding variables in this study. These variables include women’s age (15–24, 25–34, 35–49 years), age at marriage (<18, ≥18 years), birth order (<3, ≥3), place of residence (urban, rural), caste (Scheduled Caste [SC]/Scheduled Tribe [ST], Other Backward Classes [OBC], others), religion (Hindu, Muslim, others), educational level of women and that of partner (illiterate, primary, secondary, higher), media exposure (yes, no), wealth quintile and region. Exposure to mass media was measured by the frequency of reading newspapers and magazines, listening radio, and watching television and then divided into two groups: ‘no’ when none of these three media were accessed and ‘yes’ when at least any one media was accessible to the woman. Based on the ownership of household assets, housing characteristics and access to housing and sanitation facilities, household wealth quintile was estimated and categorized into five quintiles from 1 (poorest) to 5 (richest), where each quintile represents 20% of the total respondents. Further, the states and union territories of India were classified into six regions based on geographical surroundings and cultural similarities to explore the regional variation in utilization of maternal healthcare [5].

### Statistical analyses

Descriptive statistics were carried out to understand the distribution of the samples by the key predictor, covariates, and outcome variables. Bivariate percentage distribution was estimated to assess the prevalence of maternal healthcare services (full ANC, institutional delivery, and PNC within 48 hours) by the independent variables, and the differences were later tested by Pearson’s chi-square statistic. The sample weight was used for estimating percentage distribution. Finally, multivariate binary logistic regression models were applied to examine the crude and adjusted association between women’s autonomy and utilization of maternal healthcare services. The regression results were presented by the estimated odds ratio (OR) with 95% confidence interval (CI). All the statistical analyses were performed using STATA version 14.0 (StataCorp LP, College Station, TX, USA).

## Results

### Participant’s characteristics

Of the 32,698 respondents, 23.5% currently married women received full antenatal care, 82.5% delivered babies in health institutions, and 66.3% had PNC check-ups by skilled personnel within 48 hours of delivery (Table 1).

**Table 1:** Descriptive statistics of the study participants (n = 32,698).

Variables	Frequency (n)	Percentage (%)
**Independent variables**		
Decision-making autonomy		
Low	5,325	17.6
Medium	9,106	28.8
High	18,267	53.6
Women’s age (Years)		
15–24	10,217	34.3
25–34	18,763	56.4
35–49	3,718	9.3
Age at marriage (Years)		
<18	11,471	38.2
≥18	20,672	61.8
Birth order		
<3	21,374	68.5
≥3	11,324	31.5
Place of residence		
Urban	8,555	31.0
Rural	24,143	69.0
Caste		
SC/ST	12,225	31.1
OBC	12,672	47.0
Others	6,101	21.9
Religion		
Hindu	23,637	79.0
Muslim	5,354	16.3
Others	3,707	4.8
Education level		
No education	9,002	26.1
Primary	4,409	12.8
Secondary	15,543	48.0
Higher	3,744	13.1
Partner’s education		
No education	5,397	16.1
Primary	4,529	13.9
Secondary	17,985	54.5
Higher	4,699	15.5
Media exposure		
No	7,984	23.4
Yes	24,714	76.6
Wealth quintile		
Poorest	7,438	21.8
Poorer	7,326	20.7
Middle	6,693	20.5
Richer	5,868	18.9
Richest	5,373	18.2
Region		
North	6,752	13.1
Central	8,335	23.8
East	6,364	24.1
North East	4,460	3.6
West	2,963	15.0
South	3,824	20.4
**Outcome variables**		
Full ANC		
No	25,965	76.5
Yes	6,733	23.5
Institutional delivery		
No	6,841	17.5
Yes	25,764	82.5
PNC care within 48 hours		
No	11,734	33.7
Yes	20,964	66.3

Among the total study participants, 53.6% women had a high overall autonomy in the household, 28.8% had a medium level of autonomy, and 17.6% had almost no say in the decision-making. Over half of the women (56.4%) were younger in the age group of 15 to 24 years. About 38.2% women reported that they got married before completing 18 years of age. Almost one-third women (31.5%) had parity of three or more. Majority of women were residing in rural areas (69.0%), belonged to OBC category (47.0%) and believed in Hindu religion (79.0%). More than one-fourth of the women (26.1%) had no formal education, while 16.1% of their partners were illiterate. About 23.4% women had no access to mass media (newspaper/magazine, radio, or television). It is reported that 21.8% women were from the poorest wealth quintile group, while 18.2% were from the richest quintile. The highest proportion of respondents was from the eastern region (24.1%), followed by south (20.4%) and western region (15.0%).

### Utilization of maternal healthcare services by predictor variables

Significant differences were observed in utilization of maternal healthcare services (full ANC, institutional delivery, and PNC within 48 hours of delivery health personnel) by the level of autonomy and socio-demographic characteristics (Table 2). The utilization of maternal healthcare was higher among women having a high level of autonomy compared to those who had low involvement in household decision-making. The proportion of maternal healthcare was lower among the women aged 35–49 years, women who got married before 18 years of age, women with birth order above three. The women residing in rural areas and who belonged to SC/ST social groups received fewer maternity services. It was found that access to institutional delivery and PNC care was higher among Hindu women. Similarly, women with a high level of education and access to mass media had a higher proportion of maternal healthcare services. Low use of maternity care was also found among the women whose husbands were illiterate. The study also observed that the utilization of maternity services was increasing with the level of household wealth. Utilization of maternal healthcare also varied across geographical regions. The use of all three maternity care was highest in the southern region. The coverage of full ANC was lowest in the central region (8.7%), while low use of institutional delivery and PNC check-ups was found in the northeast region.

**Table 2:** Distribution of maternal healthcare services by the socio-demographic characteristics.

Variables	Full ANC		Institutional delivery		PNC care	
	Percentage	P-value	Percentage	P-value	Percentage	P-value
Decision-making autonomy		0.000		0.001		0.000
Low	19.2		80.3		59.9	
Medium	22.7		83.1		67.4	
High	25.2		82.9		67.9	
Women’s age (Years)		0.000		0.000		0.000
15–24	24.0		85.8		67.8	
25–34	24.1		82.6		66.7	
35–49	17.7		69.7		59.0	
Age at marriage (Years)		0.000		0.000		0.000
<18	15.8		75.5		59.8	
≥18	28.3		87.0		70.7	
Birth order		0.000		0.000		0.000
<3	28.3		88.9		71.2	
≥3	13.0		68.4		55.8	
Place of residence		0.000		0.000		0.000
Urban	34.3		90.5		74.4	
Rural	18.6		78.9		62.7	
Caste		0.000		0.000		0.000
SC/ST	19.9		77.4		62.9	
OBC	23.5		83.7		66.5	
Others	28.6		87.6		71.9	
Religion		0.000		0.000		0.000
Hindu	23.6		83.9		66.7	
Muslim	19.6		74.7		62.1	
Others	33.9		85.1		75.4	
Education level		0.000		0.000		0.000
No education	9.0		65.0		52.5	
Primary	16.3		76.8		62.2	
Secondary	28.3		89.5		71.3	
Higher	41.7		97.3		79.8	
Partner’s education		0.000		0.000		0.000
No education	10.8		63.1		51.1	
Primary	16.2		74.5		59.2	
Secondary	25.4		86.6		69.5	
Higher	36.7		95.3		77.5	
Media exposure		0.000		0.000		0.000
No	7.8		64.1		49.1	
Yes	28.2		88.1		71.6	
Wealth quintile		0.000		0.000		0.000
Poorest	7.6		61.7		48.9	
Poorer	14.7		78.1		61.0	
Middle	24.2		87.3		69.6	
Richer	33.0		92.3		74.7	
Richest	41.6		96.6		80.8	
Region		0.000		0.000		0.000
North	22.3		86.0		71.0	
Central	8.7		73.7		59.9	
East	12.8		73.2		57.0	
North East	18.7		72.2		57.9	
West	34.9		91.0		74.9	
South	46.5		97.1		77.1	

Note: P-value: Significance level of chi-square statistic.

### Effects of women’s autonomy on utilization of maternal healthcare services

The results of unadjusted and adjusted logistic regression models for assessing the association between women’s autonomy and utilization of maternal healthcare services are presented in Table 3. We estimated the net effect of women’s autonomy on utilization of maternal healthcare by controlling for several important socio-demographic factors including women’s age, age at marriage, birth order, place of residence, caste, religion, women’s education, partner’s education, exposure to mass media, wealth index, and geographical region. Results from Table 3 indicate that women’s autonomy is a significant predictor of maternity care in India as the odds of maternal healthcare increased with increasing the level of autonomy. Crude analysis reveals that women who had high decision-making autonomy were more likely to receive full ANC during pregnancy (UOR: 1.57, 95% CI: 1.45–1.71), institutional delivery (UOR: 1.12, 95% CI:1.04–1.21), and PNC within 48 hours of delivery (UOR: 1.40, 95% CI: 1.31–1.49) compared to those who had a low autonomy in the household. After adjusting for socio-demographic characteristics, the net effect of women’s autonomy on access to ANC and PNC care remained significant. Women with high autonomy had 37% and 33% greater likelihood of using ANC (AOR: 1.37, 95% CI: 1.25–1.50) and PNC care (AOR: 1.33, 95% CI: 1.24–1.42) respectively than women with low autonomy. However, the adjusted analysis did not portray any significant relationship between women’s autonomy and institutional delivery.

**Table 3:** Binary logistic regression models for maternal healthcare service utilization in India.

Variables	Full ANC		Institutional delivery		PNC care	
	COR (95% CI)	AOR (95% CI)	COR (95% CI)	AOR (95% CI)	COR (95% CI)	AOR (95% CI)
Decision-making autonomy						
Low ^®^						
Medium	1.33 (1.21–1.45)†	1.15 (1.04–1.28)†	1.17 (1.08–1.27)†	1.03 (0.94–1.14)	1.35 (1.26–1.45)†	1.27 (1.17–1.36)†
High	1.57 (1.45–1.71)†	1.37 (1.25–1.50)†	1.12 (1.04–1.21)†	1.05 (0.96–1.14)	1.40 (1.31–1.49)†	1.33 (1.24–1.42)†
Women’s age (Years)						
15–24 ^®^						
25–34	1.09 (1.03–1.17)†	1.07 (0.99–1.15)	0.80 (0.75–0.85)†	0.98 (0.90–1.06)	0.98 (0.93–1.02)	1.02 (0.96–1.08)
35–49	0.84 (0.76–0.93)†	1.05 (0.93–1.20)	0.43 (0.40–0.47)†	0.90 (0.80–1.02)	0.72 (0.66–0.77)†	0.98 (0.89–1.08)
Age at marriage (Years)						
<18 ^®^						
≥18	2.20 (2.07–2.34)†	1.27 (1.18–1.37)†	1.92 (1.82–2.03)†	1.12 (1.05–1.20)†	1.65 (1.57–1.72)†	1.15 (1.09–1.22)†
Birth order						
<3 ^®^						
≥3	0.42 (0.39–0.45)†	0.74 (0.68–0.80)†	0.29 (0.28–0.31)†	0.56 (0.52–0.61)†	0.51 (0.49–0.53)†	0.75 (0.70–0.80)†
Place of residence						
Urban ^®^						
Rural	0.49 (0.46–0.52)†	0.89 (0.83–0.96)†	0.39 (0.36–0.42)†	0.87 (0.80–0.96)†	0.58 (0.55–0.62)†	0.98 (0.92–1.05)
Caste						
SC/ST ^®^						
OBC	1.09 (1.02–1.16)†	0.84 (0.78–0.90)†	1.82 (1.72–1.94)†	1.21 (1.12–1.30)†	1.28 (1.22–1.35)†	1.00 (0.94–1.06)
Others	1.63 (1.52–1.76)†	1.04 (0.95–1.14)	2.29 (2.11–2.48)†	1.19 (1.08–1.32)†	1.71 (1.60–1.83)†	1.09 (1.01–1.18)*
Religion						
Hindu ^®^						
Muslim	0.83 (0.77–0.89)†	0.86 (0.78–0.94)†	0.65 (0.59–0.69)†	0.58 (0.52–0.63)†	0.92 (0.87–0.98)*	0.91 (0.85–0.99)*
Others	1.22 (1.13–1.33)†	0.99 (0.89–1.10)	0.46 (0.42–0.50)†	0.52 (0.46–0.58)†	0.76 (0.71–0.81)†	0.88 (0.80–0.97)†
Education level						
No education ^®^						
Primary	1.87 (1.68–2.10)†	1.27 (1.11–0.43)†	1.62 (1.49–1.75)†	1.15 (1.05–1.26)†	1.40 (1.29–1.50)†	1.08 (1.00–1.18)*
Secondary	3.70 (3.40–4.10)†	1.54 (1.38–1.72)†	3.85 (3.61–4.09)†	1.52 (1.39–1.66)†	2.17 (2.06–2.29)†	1.17 (1.09–1.26)†
Higher	6.65 (6.02–7.34)†	1.75 (1.52–2.02)†	15.93 (13.36–18.99)†	2.68 (2.17–3.31)†	3.79 (3.46–4.15)†	1.27 (1.12–1.44)†
Partner’s education						
No education ^®^						
Primary	1.66 (1.47–1.88)†	1.11 (0.96–1.28)	1.61 (1.47–1.75)†	1.18 (1.07–1.30)†	1.39 (1.29–1.51)†	1.15 (1.05–1.25)†
Secondary	2.76 (2.50–3.04)†	1.09 (0.96–1.23)	3.31 (3.09–3.54)†	1.33 (1.22–1.45)†	2.07 (1.95–2.21)†	1.18 (1.09–1.27)†
Higher	4.64 (4.16–5.18)†	1.11 (0.96–1.29)	9.79 (8.61–11.14)†	1.66 (1.41–1.95)†	3.44 (3.15–3.75)†	1.24 (1.10–1.38)†
Media exposure						
No ^®^						
Yes	4.20 (3.84–5.59)†	1.49 (1.33–1.67)†	3.53 (3.33–3.73)†	1.25 (1.16–1.35)†	2.40 (2.27–2.52)†	1.31 (1.23–1.40)†
Wealth quintile						
Poorest ^®^						
Poorer	2.00 (1.79–2.23)†	1.20 (1.06–1.36)†	1.88 (1.75–2.01)†	1.35 (1.24–1.47)†	1.45 (1.36–1.54)†	1.17 (1.08–1.26)†
Middle	3.41 (3.07–3.79)†	1.44 (1.26–1.63)†	3.61 (3.33–3.91)†	1.83 (1.65–2.03)†	2.26 (2.11–2.42)†	1.49 (1.36–1.62)†
Richer	5.14 (4.64–5.71)†	1.82 (1.58–2.09)†	6.54 (5.92–7.21)†	2.55 (2.24–2.91)†	2.95 (2.74–3.18)†	1.73 (1.56–1.91)†
Richest	7.44 (6.71–8.24)†	2.40 (2.07–2.80)†	14.65 (12.77–16.82)†	4.03 (3.37–4.82)†	4.46 (4.11–4.84)†	2.27 (2.01–2.56)†
Region						
North ^®^						
Central	0.38 (0.35–0.42)†	0.53 (0.48–0.59)†	0.54 (0.50–0.59)†	0.81 (0.74–0.90)†	0.62 (0.58–0.67)†	0.89 (0.83–0.97)†
East	0.48 (0.43–0.52)†	0.81 (0.72–0.90)†	0.51 (0.47–0.56)†	1.01 (0.91–1.12)	0.58 (0.54–0.62)†	0.97 (0.90–1.06)
North East	0.89 (0.81–0.97)*	1.12 (0.99–1.26)	0.35 (0.32–0.39)†	0.65 (0.58–0.74)†	0.47 (0.44–0.51)†	0.66 (0.60–0.73)†
West	1.82 (1.67–2.01)†	1.97 (1.77–2.18)†	1.52 (0.32–1.73)†	1.48 (1.27–1.71)†	1.02 (0.93–1.12)	1.12 (1.01–1.25)*
South	2.86 (2.63–3.12)†	2.79 (2.53–3.08)†	6.29 (5.15–7.69)†	5.45 (4.34–6.85)†	1.26 (1.14–1.38)†	1.22 (1.10–1.35)†

Note:

^®^ Reference category,

^†^ *p*<0.01,

* *p*<0.05;

*COR*, unadjusted odds ratio; *AOR*, adjusted odds ratio; *CI*, confidence interval.

The results also reveal that women who got married at 18 years or later had a greater probability of accessing maternal care services as compared to those married before 18 years. Women living in rural areas and having a parity of 3 or more were associated with a lower likelihood of utilization of maternal healthcare services. Women who belonged to OBC had lower odds of full ANC care and higher odds of institutional delivery than the SC/ST caste category. Similarly, women of ‘others’ caste group had more chances of getting institutional delivery and PNC care compared to SC/ST category. Muslim women were associated with a lower likelihood of receiving maternity care services than that of Hindus. Education of both women and partner was found to be positively associated with all three components of maternity care. Access to mass media was also found to be an important factor for maternal care utilization. Exposure to mass media increased the odds of getting different maternity services by 25% to 49%. The probability of maternity care increased with an increasing level of household wealth. Compared to women from the poorest quintile group, women of the richest quintile had almost two-fold higher likelihood of getting ANC and PNC care and four-fold of institutional delivery. The study also found that geographical region had a significant influence on maternity care utilization. Women from the south and west region were more likely and women from the central region were less likely to receive maternal services than the north region.

## Discussion

The present study has examined the association between women’s decision-making autonomy and the utilization of maternal healthcare services using nationally representative samples. It is observed that a major proportion currently married women in India had a high (53.6%) and medium (28.8%) level of overall autonomy regarding own healthcare, household purchases, spending of money, and freedom of movement. The study found that women’s autonomy is a significant predictor of maternity care in India. There is a strong association between women’s decision-making autonomy and the use of ANC care during pregnancy and PNC care within 48 hours of delivery even after adjusting for socio-demographic variables. Our findings are consistent with the previous studies conducted in Bangladesh [10], Ethiopia [12, 19], Nepal [11], and sub-Saharan African countries [20]. Maitra (2004) illustrated that a woman’s control over household resources has a significant positive effect on both the demand for postnatal care and the probability of having delivery in a health institution, and the demand for antenatal care is also significantly higher if the woman has a say in decision-making regarding health care [21]. Similarly, another study observes that women who participate in the decision-making of households are more likely to have a higher level of autonomy on health care, which might reduce their reproductive behavior risks [22]. It is argued that the more autonomous women are, the greater influence they will have within their households and in society and the more likely they are to visit a health facility for personal and child healthcare [23]. In a field-based study of an Indian city, Bloom et al. [8] explored that the influence of women’s autonomy on the use of healthcare appears to be as important as other known determinants, such as education. In contrast to other studies [11, 12], the results of this study show that there is no significant association between women’s autonomy and institutional delivery in the adjusted analysis. However, this result of the present study is consistent with the findings of some studies conducted in Nepal [24] and India [3] that decision-making autonomy does not necessarily affect the likelihood of having institutional deliveries.

Other socio-demographic characteristics of women at individual and household level have significant effects on maternal healthcare services. Similar to other studies [17, 25–27], the present study finds that higher educational attainment of both women and their husbands, exposure to mass media, household wealth were positively associated with utilization of maternal healthcare services. Several studies confirm that education enhances the autonomy of women to make decisions and the capability to use quality health care inputs that offer better care [3, 28]. Likewise, exposure to mass media, for instance, reading newspapers, watching television, and listening to radio facilitates in increasing awareness among the people regarding maternity care and access to health care information [17]. In contrast, women with high parity, early marriage (before 18 years), residing in rural areas, and belonging to SC/ST caste groups were associated with a decreased likelihood of maternity care utilization. These findings are in tune with several studies of India and elsewhere [27–29]. Further, geographical differences had a significant impact on utilization of maternal healthcare. In agreement with the previous study [3], the present study also showed that women residing southern region were more likely to receive adequate ANC, delivery institutional delivery, and PNC care. South India has a higher level of women autonomy and better health infrastructure; thereby, women are better able to obtain available pregnancy care services than the north region [3, 22].

The present study has some limitations. One of the limitations is the cross-sectional design of this study; thereby, we could not be able to examine the causal relationship between women’s autonomy and maternal healthcare. Secondly, the study utilized the data that collected information on maternity care services retrospectively up to five years in the past. The possibility of recall bias thus cannot be ignored. Further, women’s autonomy was measured by using four dimensions of decision-making. However, there might be other potential aspects of autonomy that could not be incorporated due to limited information. Nonetheless, some important indicators of women’s autonomy, such as healthcare, household purchase, financial spending, and freedom of movement that have marked impact on maternity care, were included in this study. Another aspect of this study is that it provides an understanding of the influence of women’s autonomy on utilization of maternal healthcare using nationally representative, population-based, large-scale samples. Therefore, an effort has been made in this study to identify the broader areas necessitating effective policy interventions for the improvement of maternal healthcare service utilization.

## Conclusion

This study has highlighted that the utilization of maternal healthcare services among women of reproductive age group in India is still inadequate. A substantial proportion of women did not receive WHO-recommended adequate antenatal care, institutional delivery, and PNC care after delivery in this country. We observed that women’s autonomy in seeking healthcare, making household purchases, spending money, and deciding movement is an important predictor of the use of maternal healthcare services. It is, therefore, recommended to focus on the need for comprehensive strategies involving improvement of women’s autonomy along with expansion of education, awareness generation regarding the importance of maternity care, and enhancing public health infrastructure to ensure higher utilization of maternal healthcare services that would eventually reduce maternal mortality.
